# Structural Stability and Deformation of Solvated Sm@*C*_*2*_(45)-C_90_ under High Pressure

**DOI:** 10.1038/srep31213

**Published:** 2016-08-09

**Authors:** Jinxing Cui, Mingguang Yao, Hua Yang, Ziyang Liu, Shijie Liu, Mingrun Du, Quanjun Li, Ran Liu, Tian Cui, Bingbing Liu

**Affiliations:** 1 State Key Laboratory of Superhard Materials, Jilin University, No. 2699 Qianjin Street, Changchun 130012, P.R. China; 2 College of Materials Science and Engineering, China Jiliang University, No. 258 Xueyuan Street, Hangzhou 310018, P.R. China

## Abstract

Solvated fullerenes recently have been shown to exhibit novel compression behaviors compared with the pristine fullerenes. However, less attention has been focused on the large cage endohedral metallofullerenes. Here, we have firstly synthesized solvated Sm@C_90_ microrods by a solution drop-drying method, and then studied the transformations under high pressure. The pressure-induced structural evolutions of Sm@C_90_ molecules both undergo deformation and collapse. The band gaps of both samples decrease with increasing pressure. The trapped Sm atom plays a role in restraining the compression of the adjacent bonds. The solvent plays a role in protecting Sm@C_90_ against collapse in the region of 12–20 GPa, decreasing and postponing the change of band gap. Above 30 GPa, the carbon cages collapse. Released from 45 GPa, the compressed solvated Sm@C_90_ forms a new ordered amorphous carbon cluster (OACC) structure with metal atoms trapped in the units of amorphous carbon clusters, which is different from the OACC structure formed by compressing solvated C_60_ and C_70_. This discovery opens the door for the creation of new carbon materials with desirable structural and physical properties when suitable starting materials are selected.

Solvated fullerenes which are composed of fullerene molecules separated by solvent molecules are receiving considerable attention due to high stability, tunable metrics and functionality^1^. The interactions between fullerenes and solvents reduce the symmetry of fullerene molecules, allowing electronic transitions that are forbidden in pristine fullerenes and inducing a strong photoluminescence, which gives potential applications in the optical field^2^,^3^. High-pressure study on several solvated C_60_ and C_70_ with different solvents and structures has attracted much attention recently. Some new phenomena have been observed. For example, C_60_{Ni(nPr_2_dtc)_2_} and C_60_{Cu(nPr_2_dtc)_2_} show the pressure-induced charge transfer from the organic donor to the fullerene acceptor near 2 GPa^4^. Fc solvated C_60_ and C_70_ show a reversible pressure-induced polymerization^5^,^6^. C_60_*m-xylene can form an ordered amorphous carbon cluster (OACC) structure under high pressure, which exhibits high hardness and can indent diamond^1^. The intercalated solvent acts as bridge and spacer to preserve the stability of the building blocks, deformed or amorphized C_60_ molecules^7^. Our latest work reported that compressed C_70_*m-xylene can also form a new OACC structure by undergoing a different phase transition routine due to the anisotropic deformation of C_70_ molecules under pressure^8^, which suggests that the size and symmetry of the carbon cage can influence the phase transition. If the OACC structure contains metal atoms, it will be possible to create new intercalated sp^3^ amorphous carbon materials with excellent physical properties^9^,^10^. Compared with C_60_ and C_70_, high endohedral metallofullerenes (EMFs) have larger carbon cage, lower molecular symmetry and metal atoms trapped inside. EMFs are expected to be a good building block for constructing new carbon structures and have put forward new ideas for the design of new materials. It is still unknown that whether solvated EMFs can also form OACC structures with high hardness, how EMFs molecules deform, and what solvent molecule affection is under high pressure. Meanwhile, it is also an interesting topic to study the influence of embedded metal atom on structural deformation, mechanic and electronic properties of solvated EMFs under high pressure.

EMFs are the fullerenes with metal atom trapped inside, in which electron transfer from the metal atom to the carbon cage takes place^11^. EMFs thus possess unique structures and electronic properties which are different from hollow fullerenes^12^, and have been attracting intensive research interest. With these the properties, EMFs exhibit many potential applications in biomedicine and nanomaterial science^13^,^14^, such as optoelectronic devices^15^ and molecular electronics^16^. Numerous EMFs have been produced since Heath *et al*. reported the discovery of La@C_60_ by mass spectroscopy^17^. However, the low production efficiency and the isomer mixtures of EMFs have hindered studies of the intrinsic structures and properties of EMFs. Recently, Yang *et al*. have isolated and characterized four isomers of Sm@C_90._ Among them, Sm@*C*_*2*_(42)-C_90_ is experimentally the most abundant isomer, and computationally the most stable configuration along with the largest HOMO-LUMO gap^18^. As yet, the fundamental vibrational spectra and physical properties of Sm@*C*_*2*_(42)-C_90_ remain to be implemented. This material offers an ideal model to study the structural stability and deformation under high pressure.

In this work, we have prepared solvated EMF (Sm@C_90_*m-xylene) by a solution drop-drying process. Then, the measured vibrational spectra of Sm@C_90_ at ambient conditions have been assigned with the help of theoretical calculation. The structural stability and deformation of Sm@C_90_ under high pressure have been studied by Raman and IR spectroscopy. The roles of the solvent on the deformation of Sm@C_90_ upon compression have been studied by comparative experiments on both solvated and pristine Sm@C_90_. For the first time, we report a new OACC structure with metal atoms trapped in the units of carbon clusters, which extends the OACC structure to EMFs. It is highly promising to further produce new carbon materials with unique structure and outstanding physical properties using this method.

## Results and Discussion

**Figure 1:**
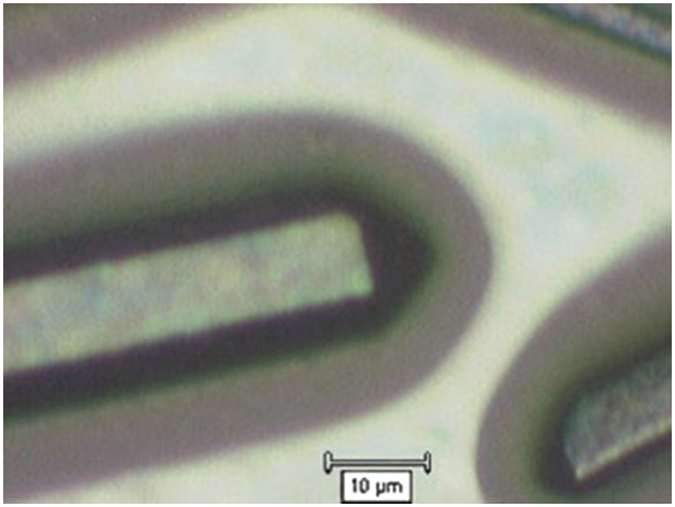
The image of solvated Sm@C_90_ microrods.

**Figure 2:**
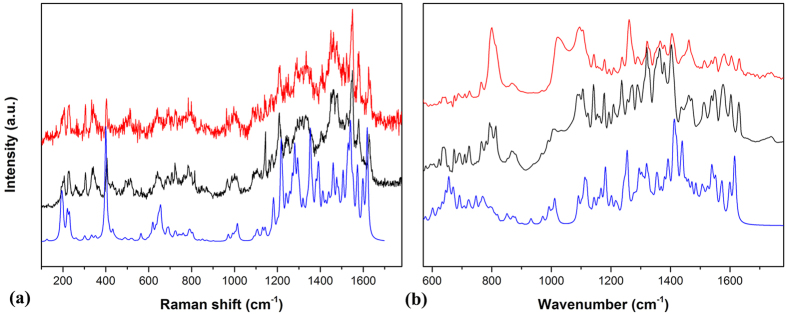
(**a**) Experimental Raman spectra of solvated (red) and pristine (black) Sm@C_90_ and calculated (blue) Raman spectra of Sm@C_90_ at ambient pressure. (**b**) Experimental IR spectra of solvated (red) and pristine (black) Sm@C_90_ and calculated (blue) IR spectra of Sm@C_90_ at ambient pressure.

**Figure 3:**
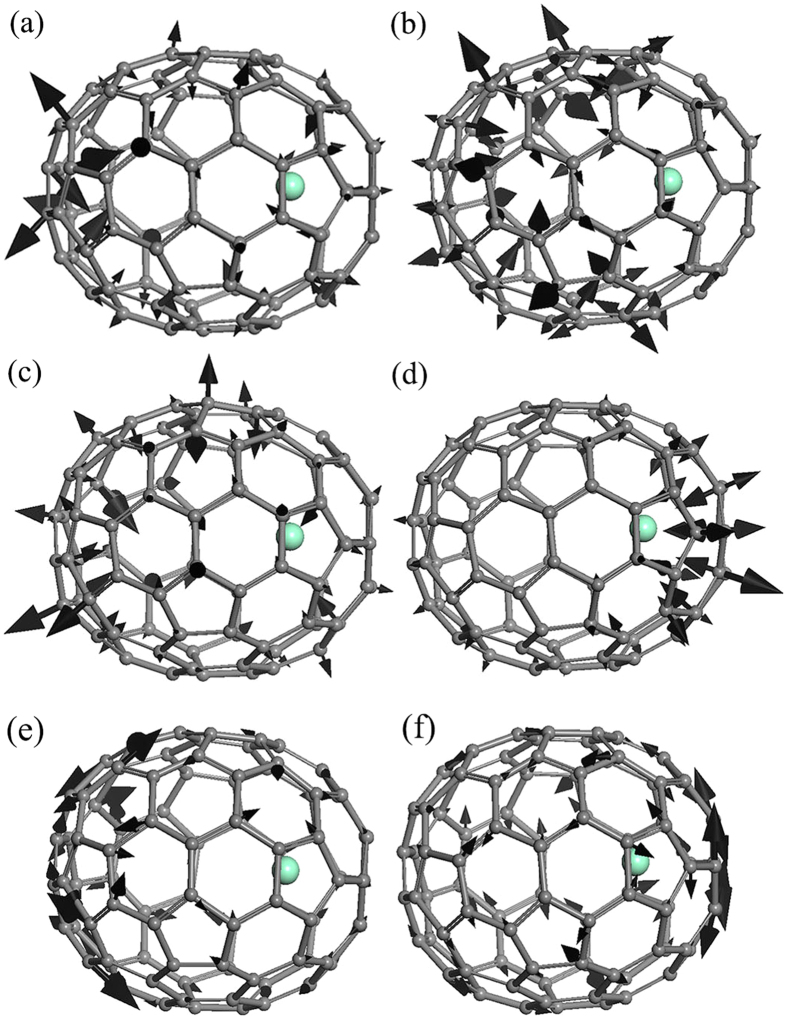
The eigenvectors of IR-active vibrational modes at (**a**) 640, (**b**) 709, (**c**) 724, (d) 799 cm^−1^ and Raman-active vibrational modes at (**e**) 1221, (**f**) 1487 cm^−1^.

Figure 1 shows the optical image of solvated Sm@C_90_*m-xylene microrods on a glass substrate. The diameter distribution of these rods is 10–20 μm. The Raman and IR spectra of solvated and pristine Sm@C_90_ at ambient conditions are displayed in Fig. 2. The spectra of Sm@C_90_*m-xylene show vibration modes similar to those of pristine Sm@C_90_, suggesting only a week van der Waals interaction between the solvent and Sm@C_90_ molecules. The IR spectrum of solvated Sm@C_90_ shows clear absorption peaks from the solvent *m*-xylene, confirming that *m*-xylene molecules were intercalated in the Sm@C_90_ crystals. No reference data on the assignment of the vibrational modes of Sm@C_90_ exists. To understand the origin of these vibrational modes we have calculated the Raman and IR spectra for Sm@C_90_ with DMOL^3^ at ambient conditions. Based on the simulation, the vibrational spetra can be roughly divided into three regions. (1) The bands from 200 to 500 cm^−1^ are attributed to the radial breathing vibrations of the carbon cage. (2) The bands between 500 and 800 cm^−1^ are from C-C/C = C radial banding vibrations. (3) The bands from 800 to 1700 cm^−1^ are related with C-C/C = C tangential stretching vibrations on spherical surface. Figure 3 demonstrates the eigenvectors of some IR-active vibrational modes at 640, 709, 724 and 799 cm^−1^ and some Raman-active vibrational modes at 1221 and 1487 cm^−1^.

### Raman spectroscopy

**Figure 4:**
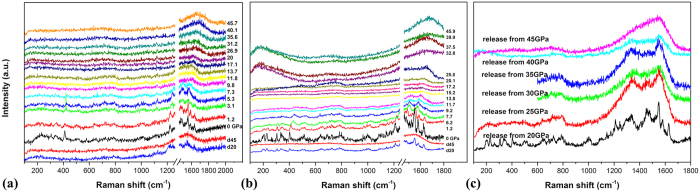
(**a**) Raman spectra of pristine Sm@C_90_ under high pressure and released from 45 and 20 GPa (marked “d45” and “d20”). (**b**) Raman spectra of solvated Sm@C_90_ under high pressure and released from 45 and 20 GPa (marked “d45” and “d20”). (**c**) Raman spectra of solvated Sm@C_90_ decompressed from different pressures.

Figure 4 shows the Raman spectra of pristine and solvated Sm@C_90_ under high pressure and decompressed from different pressures to ambient pressure. The Raman spectroscopic evolutions of the two samples are similar upon compression and decompression. From the recorded spectra under high pressure, we can see that the Raman peaks shift, broaden and weaken with increasing pressure. Above 30 GPa, most Raman peaks disappear, except for a broad, intensive band in the 1600–1800 cm^−1^ region and a weak broad band in the 600–800 cm^−1^ region can be observed, suggesting the collapse of Sm@C_90_ molecules. In order to investigate the structural stability of the materials after compression, we also performed Raman measurements on the released samples. The Raman spectra of both samples decompressed from 20 GPa are similar to those of samples at ambient pressure, indicating the reversibility of the deformation of Sm@C_90_ molecules within the compressed pressure as high as 20 GPa. In the Raman spectrum of solvated Sm@C_90_ decompressed from 40 GPa, most modes disappeared except for two broad bands in the 1200–1600 cm^−1^ and a weak broad band in the 600–800 cm^−1^ region, shown in Fig. 4c. In the spectra of both samples decompressed from 45 GPa, only one broad band around 1600 cm^−1^ can be observed, suggesting the collapse of Sm@C_90_ cages can be quenched to ambient conditions. Such Raman features supply the evidences that the high pressure phase contains both sp^2^ and sp^3^ bonds^19^,^20^ and Sm@C_90_ molecules undergo irreversible collapse above 45 GPa. It is remarkable that the irreversible collapse pressure of solvated EMFs is higher than those of solvated C_60_^1^ and C_70_^8^ of which the irreversible collapse occurs at about 35 GPa. This indicates that the large carbon cage of Sm@C_90_ is more stable against compression.

### IR spectroscopy

**Figure 5:**
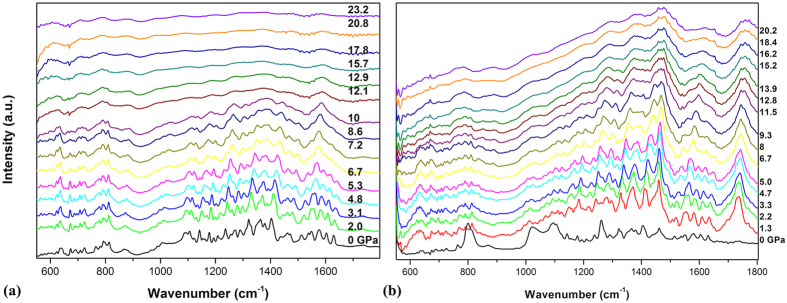
(**a**) IR spectra of pristine Sm@C_90_ under high pressure. (**b**) IR spectra of solvated Sm@C_90_ under high pressure.

Figure 5 shows the IR spectra of pristine and solvated Sm@C_90_ under high pressure. With increasing pressure, the intensities of major peaks in IR spectra of pristine Sm@C_90_ decrease and broaden significantly above 12 GPa, indicating that the carbon cages start to lose the cage structure and collapse. However, for the solvated Sm@C_90_, the dominant IR peaks remain visible and distinguishable up to 20 GPa, suggesting that Sm@C_90_ molecules still preserved their carbon cages. This indicates that the solvent molecules play a protection role for Sm@C_90_ molecules against the compression in this pressure region. Further increasing pressure to above 30 GPa, the carbon cages collapse (shown in Fig. 4).

**Figure 6:**
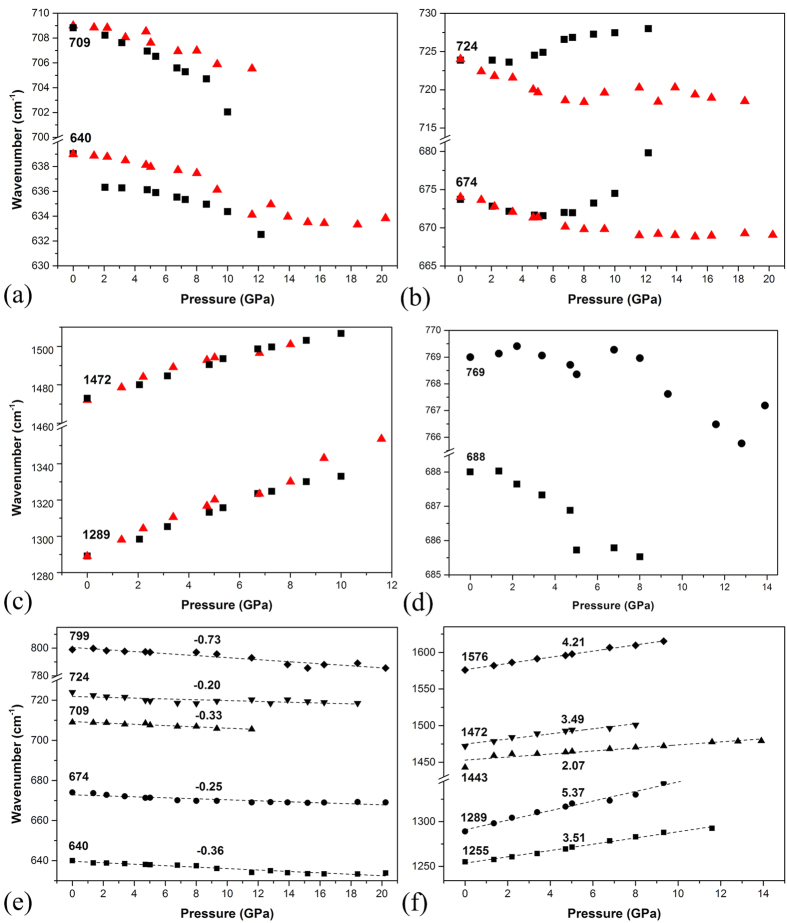
(**a**) Frequencies of IR-active vibrational modes at 709, 640, (**b**) 724, 674, (**c**) 1472 and 1289 cm^−1^ for pristine (black square) and solvated (red triangle) Sm@C_90_ as a function of pressure. (**d**) IR-active vibrational modes of solvent (*m*-xylene) at 688 and 769 cm^−1^ as a function of pressure. (**e**) The C-C/C = C radial bending vibrations and (**f**) the C-C/C = C tangential stretching vibrations of solvated Sm@C_90_ as functions of pressure. The pressure coefficients are shown near the fitted lines.

The pressure evolution for the frequencies of the IR-active vibrational modes have been fitted by using Lorentzian functions. It can be seen that some peaks exhibit obvious either blue or red shifts with increasing pressure. The pressure-induced blue shift of the vibrational modes may result from the shrinkage of bond length with increasing pressure. The pressure-induced red shift of the vibrational modes may be due to pressure-strengthened intermolecular attracting force, which causes an extension of the corresponding bonds and changes of corresponding bond angles. A pressure-induced red shift of IR-active vibrational modes in the frequency region from 600 to 800 cm^−1^ in C_60_ and C_70_ has been observed in earlier high pressure studies^21^,^22^. Further observation on both samples of pristine and solvated Sm@C_90_ gave that the pressure coefficients of the blue-shifted C-C/C = C tangential stretching vibrational modes such as 1472 and 1289 cm^−1^ are similar, (shown in Fig. 6c). The pressure coefficients of the C-C/C = C radial banding vibrational modes showing red shift from solvated Sm@C_90_ are smaller than those from pristine Sm@C_90_, such as the modes at 640 and 709 cm^−1^ (shown in Fig. 6a). The smaller pressure coefficients may result from the intercalation effect of the solvent molecules in the formed solvated crystals, in which the solvent may hinder Sm@C_90_ molecules from approaching and thus decrease the pressure-induced strengthen of the intermolecular interactions. There are also obvious differences in the pressure coefficients of some vibrational modes between solvated and pristine Sm@C_90_, such as the modes at 674 and 724 cm^−1^ (shown in Fig. 6b). These modes are all related to the C-C/C = C radial banding vibrations of the regions of the carbon cage far away from the Sm atom (the eigenvectors of the vibrational modes at 640, 709 and 724 cm^−1^ are shown in Fig. 3), giving us a clue that these regions of cage could be subjected to sensitive interaction from solvate molecules. The reason may be that solvate molecules are preferably located around the weak polarity regions of the carbon cage far away from the Sm atom, or there is a special interaction between Sm and cage on the basis of the encapsulation of Sm. The IR-active vibrational modes of solvent (*m*-xylene) at 688 and 769 cm^−1^ exhibit a slight red shift with increasing pressure, which may result from pressure-induced enhancement of intermolecular van der Waals force between the carbon cage and solvent molecules^23^.

Figure 6e shows the red shift of five intense C-C/C = C radial banding vibrational modes at 799, 724, 709, 674 and 640 cm^−1^ from solvated Sm@C_90_ with increasing pressure. Comparing with the pressure coefficients of these vibrational modes, we find that the closer the relevant regions of the carbon cage are to the Sm atom, the larger are the pressure coefficients of the corresponding C-C/C = C radial banding vibrational modes. For example, the vibrational mode at 724 cm^−1^, which is from the part far away from the Sm atom, exhibits a smaller pressure coefficient of −0.20 cm^−1^GPa^−1^, while the vibrational mode at 799 cm^−1^, which is from the part close to the Sm atom, exhibits a much larger pressure coefficient of −0.7 cm^−1^GPa^−1^. The nonuniform charge distribution on the carbon cage results in a stronger interaction between the Sm atom and the neighboring C atoms. Such interaction may stretch the object bonds, thus slow down the corresponding C-C/C = C radial banding vibrations, speeding up the red shift of these vibrational modes with increasing pressure. We plotted the pressure dependences of five intense C-C/C = C tangential stretching vibrational modes at 1576, 1472, 1443, 1289 and 1255 cm^−1^ from solvated Sm@C_90_ in Fig. 6f, which exhibit a blue shift with increasing pressure. Comparing with the pressure coefficients of these vibrational modes, we find the opposite rule that the closer the relevant regions of the carbon cage are to the Sm atom, the smaller are the pressure coefficients of the corresponding C-C/C = C tangential vibrational modes. For example, the vibrational mode at 1289 cm^−1^, which is from the part far away from the Sm atom, exhibits a larger pressure coefficient of 5.37 cm^−1^GPa^−1^, while the vibrational mode at 1443 cm^−1^, which is from the part close to the Sm atom, exhibits a much smaller pressure coefficient of 2.07 cm^−1^GPa^−1^. This indicates that the interaction between Sm and the near area of the carbon cage minimizes the compression of the adjacent bonds, thus restraining the changes of the corresponding C-C/C = C tangential stretching vibrations with increasing pressure. This also indicates the anisotropic deformation of Sm@C_90_ cage, in which the area of the carbon cage closer to the Sm atom deforms less and the symmetry of the carbon cage reduces. This phenomenon has been observed in our study of Sm@C_88_ under high pressure^24^.

**Figure 7:**
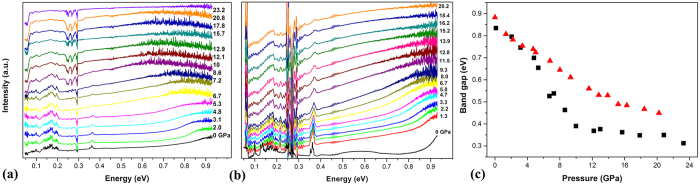
(**a**) The absorption edge of pristine Sm@C_90_ and (**b**) solvated Sm@C_90_ under high pressure. (**c**) The calculated band gaps of pristine (black square) and solvated (red triangle) Sm@C_90_ as a function of pressure.

Furthermore, to understand the pressure evolution for the electronic structure of Sm@C_90_ samples, we derived the band gaps of the two samples from their IR absorption spectra recorded under pressure. It can be seen that the absorption edges of both samples gradually downshift to lower energy with increasing pressure. The direct band gap E_g_ of the sample can be estimated from the x-axis intercept by extrapolating the linear portion of the (αhν)^2^ versus hν plot to α = 0, where α and hν are the absorption coefficient and the incident photon energy. As shown in Fig. 7c we obtained the band gaps as a function of pressure from the absorption edges of the samples. The band gap from pristine Sm@C_90_ decreases with increasing pressure, then starts to change slowly at 12 GPa. This pressure is close to that in which the carbon cages start to collapse. Thus the nolinear change of the band gap could be related to the collapse of Sm@C_90_ molecules. Similar phenomenon has been observed in C_60_^25^,^26^. The reduction of the band gaps may result from the pressure-induced deformation of Sm@C_90_ molecules and enhancement of the intermolecular interaction. The reduction in the band gap of solvated Sm@C_90_ is smaller than that of pristine sample. The pressure coefficient of the band gap from solvated sample is smaller than that from pristine sample, and varies slowly above 15 GPa, which is higher than 12 GPa for pristine sample. These phenomena may result from that solvated molecules act as spacers to protect Sm@C_90_ molecules against the deformation, and to decrease the enhancement of the intermolecular interaction, thus decreasing and postponing the change of band gap.

**Figure 8:**
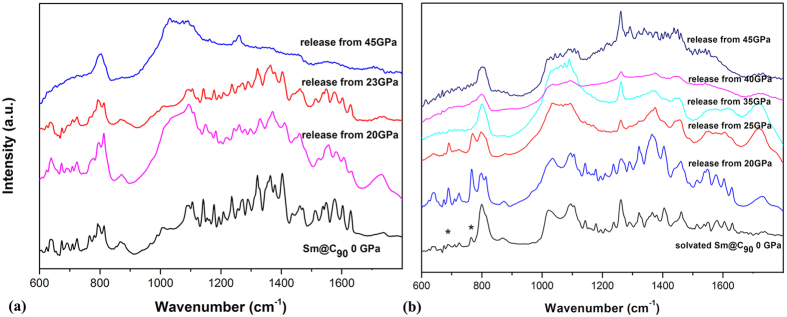
(**a**) IR spectra of pristine Sm@C_90_ released and (**b**) solvated Sm@C_90_ decompressed from different pressures to ambient pressure. The peaks from the solvent are marked with asterisks.

In order to investigate the structural stability of the materials after compression, we also measured the IR spectra of pristine and solvated Sm@C_90_ released from different pressures at ambient pressure (shown in Fig. 8). The spectra of both samples decompressed from 20 GPa preserved all the vibrational modes, and exhibit similar spectroscopic features to those of the samples at ambient pressure. This indicates the collapse of pristine Sm@C_90_ below 20 GPa is reversible. In contrast, most modes disappear in the spectra of both samples decompressed from above 30 GPa, suggesting the collapse of Sm@C_90_ molecules can be quenched. The vibrational modes from the solvent can’t be also detectable in the solvated Sm@C_90_ released from above 30 GPa. This is different from the solvated C_60_ and C_70_, in which the solvent molecules are preserved in the decompressed sample from similar pressure region^1^,^7^,^8^. That might be due to the different interactions between the carbon cages and solvent molecules under pressure and the broken fullerene fragments may react and bond with the solvent molecules.

### HRTEM and XRD

**Figure 9:**
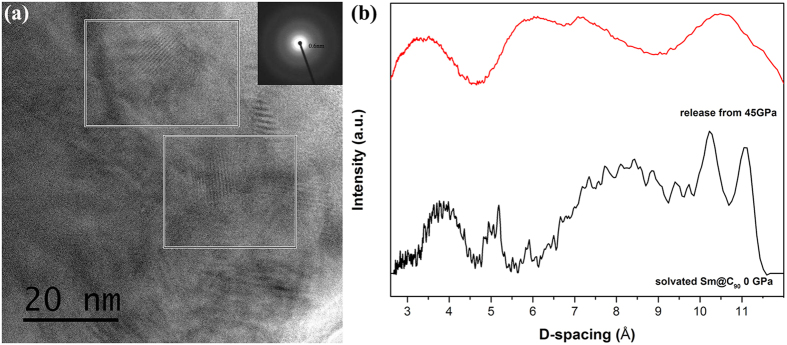
(**a**) HRTEM image and SAED pattern (inset) of solvated Sm@C_90_ decompressed from 45 GPa. (**b**) The XRD patterns of solvated Sm@C_90_ at ambient conditions (black) and decompressed from 45 GPa (red).

To determinate whether the decompressed solvated Sm@C_90_ form the OACC structure. High resolution TEM (HRTEM) and XRD are used to further study the microstructures of the decompressed samples. Figure 9 shows the HRTEM image and XRD pattern of the solvated Sm@C_90_ decompressed from 45 GPa. The HRTEM image of the sample shows a number of disordered phases and a few areas of ordered phases, which is similar to the results of decompressed solvated C_60_ and C_70_^1^,^7^,^8^. In addition, selected area electron diffraction (SAED) pattern from the ordered parts shows a weak diffracted ring with d value of around 0.6 nm. XRD pattern of released sample of solvated Sm@C_90_ shown in Fig. 9b presents several broad peaks with the profile and d values rough similarity to those of solvated Sm@C_90_ at ambient conditions. The appearance of series of broad peaks, contrasted to that of only one broad band of amorphous and nonperiodic carbon phases produced form pure fullerenes^27^,^28^, indicates that decompressed solvated Sm@C_90_ may remain the periodic arrangement. The peaks in the XRD patterns of either released or ambient samples are broad, which may be concern with large size, disorder orientation of Sm@C_90_ molecules. This high pressure phase is a new ordered amorphous carbon cluster structure (OACC), a new carbon material with the embedment of metal atoms in the units of carbon clusters and preservation of the long-range periodicity, indicating that it is possible to create new carbon materials with the potential for a huge variety of physical properties, such as light-emitting quantum dots and so on. The high compaction between solvent π-rings and great aromatic moiety of EMFs cages due to π-π interaction under high pressure may result in the covalent band formations and polymerizations between the solvent molecules and compressed or collapsed Sm@C_90_ cages, thus the periodic arrangement is retained^7^,^10^. There is no any crack on the diamond anvils after compression of solvated Sm@C_90_ in our experiments, being different from that of compressing solvated C_60_ and C_70_. For the large carbon cage and orientation disorder of Sm@C_90_, dislocations may take place between the units of amorphous carbon clusters under high pressure, reducing the hardness of the newly formed material. Our recent study on solvated C_60_ (C_60_*Fc and C_60_*NiOEP) showed that the presence of ionic carbon-metal bonding in the compressed solvated fullerenes may result in low bond-bending force constants^10^. The effect of Sm atom may also leads to the unstability of the solvent molecules and thus decreases the mechanical properties of the high pressure phase.

## Conclusions

Solvated Sm@C_90_ microrods have been prepared by a solution drop-drying method. The vibrational spectra of Sm@C_90_ at ambient conditions have been given and assigned for the first time. The compression behaviors of solvated Sm@C_90_ under high pressure have been studied and compared with the pristine Sm@C_90_ by Raman and IR spectroscopy. The pressure-induced structural evolutions of Sm@C_90_ in the two samples both undergo deformation and collapse. The trapped Sm atom plays a key role in minimizing the compression of the adjacent bonds. The band gap of pristine Sm@C_90_ decreases more slowly than that of solvated Sm@C_90_. The solvents protect Sm@C_90_ against collapse in the pressure region of 12–20 GPa, decreasing and postponing the change of band gap, while the carbon cages collapse with the pressure beyond 30 GPa. Sm@C_90_ decompressed from 45 GPa form a new OACC structure with metal atoms embedded in the units of amorphous carbon clusters. The solvents have been found to act as spacers and bridges to keep the collapsed Sm@C_90_ cages order. These studies extend the OACC structure to EMFs and open the door for the creation of new carbon materials with desirable structural and physical properties when suitable starting materials are selected.

## Methods

Solvated Sm@C_90_ microrods were prepared on a glass substrate by evaporating a saturated Sm@C_90_/*m*-xylene solution at room temperature. The samples were solvated crystals which have been characterized by IR and Raman. The solvated and pristine Sm@C_90_ were loaded separately in 100 μm diameter hole drilled in the T301 stainless steel gasket that was compressed in a diamond anvil cell (DAC). The pressure was calibrated by the ruby fluorescence technique. For the IR measurements, KBr was used as pressure medium and no pressure medium was used in the Raman measurements. The Raman spectra were recorded using a Renishaw 1000 notch filter spectrometer equipped with 514 nm exciting laser. The IR spectra was collected in transmission mode by a Bruker Vertex 80 v FTIR spectrometer and Hyperion 2000 IR microscope equipped with a nitrogen-cooled MCT detector. The microstructure of the sample decompressed from high pressure was analyzed by transmission electron microscopy (JEM-2200FS). The XRD measurement was performed at Shanghai Synchrotron Radiation Facility.

All *first-principles* calculations (including geometry optimizations and vibrational frequencies) were carried out by the DMOL^3^ method within the gradient-corrected approximation (GGA).

## Additional Information

**How to cite this article**: Cui, J. *et al*. Structural Stability and Deformation of Solvated Sm@*C*_*2*_(45)-C_90_ under High Pressure. *Sci. Rep*. **6**, 31213; doi: 10.1038/srep31213 (2016).
