# Corrigendum: Structural Stability and Deformation of Solvated Sm@C_2_(42)-C_90_ under High Pressure

**DOI:** 10.1038/srep33773

**Published:** 2016-12-14

**Authors:** Jinxing Cui, Mingguang Yao, Hua Yang, Ziyang Liu, Shijie Liu, Mingrun Du, Quanjun Li, Ran Liu, Tian Cui, Bingbing Liu

Scientific Reports 6: Article number: 31213; 10.1038/srep31213 published online: 08 09 2016; updated: 12 14 2016.

The original version of this Article contained an error in the title of the paper.

“Structural Stability and Deformation of Solvated Sm@C_2_(45)-C_90_ under High Pressure”.

should read:

“Structural Stability and Deformation of Solvated Sm@C_2_(42)-C_90_ under High Pressure”.

This has now been corrected in both the PDF and HTML versions of the Article.

